# The protection mechanism of proline from D-galactosamine hepatitis involves the early activation of ROS-eliminating pathway in the liver

**DOI:** 10.1186/s40064-015-0969-8

**Published:** 2015-04-28

**Authors:** Yoko Obayashi, Harumi Arisaka, Shintaro Yoshida, Masato Mori, Michio Takahashi

**Affiliations:** Research Institute for Health Fundamentals, Ajinomoto Co., Inc., 1-1Suzuki-cho, Kawasaki-ku, 210-8681 Kawasaki, Kanagawa Japan; Present address: 1-15-1, Kyobashi, Chuo-ku, 104-8315 Tokyo, Japan

**Keywords:** Proline, D-galactosamine, Reactive oxygen species, Antioxidation, Catalase, Glutathione

## Abstract

The oral pre-administration of proline, one on the non-essential amino acids, has been shown to effectively protect the liver from D-galactosamine (GalN)-induced liver injury and dramatically improve the survival rate. In the previous study, we reported that protective effect of proline involves the early activation of IL-6/STAT-3 pathway, an anti-inflammatory and regenerative signaling in the liver. Reactive oxygen species (ROS) are mediator of cellular injury and play an important role in hepatic damage during GalN-induced hepatitis. The aim of this study is to investigate the effect of proline on ROS-eliminating system. The activities of major ROS-detoxifying enzymes, i.e., glutathione peroxidase (GP), glutathione reductase (GR), catalase, and the level of glutathione in the liver were determined. Catalase activity was significantly upregulated in proline group from 0 to 3 h after GalN-injection, although GP and GR were downregulated during this period, compared with control group. From 6 to 12 h, the level of reduced glutathione (GSH) was significantly higher and the ratio of GSH/oxidized glutathione (GSSG) tended to be higher in proline group. Consistently with this, at 6 h, the GR activity in the proline group was significantly higher, followed with the higher tendency of GP activity at 12 h. Catalase activity was also significantly higher at 12 h. Taken together, catalase was activated at the beginning, followed with the significant activation of glutathione redox system around 6 to 12 h in proline group. These results suggest that the elimination of ROS in the liver was accelerated in proline group compared with control group at the very early stage of GalN-induced hepatitis.

## Background

It is reported that endotoxemia significantly contributes to the pathogenesis of GalN hepatitis (Grün et al. 1977), by activating inflammatory cells followed with the secretion of inflammatory mediators such as TNF-α (Jirillo et al. 2002). Activated macrophages infiltrate into the hepatic stroma and cause extensive hepatocellular necrosis (MacDonald et al. 1987). Activated inflammatory cells, e.g. hepatic macrophages, Kupffer cells, hepatic neutrophils, produce and release large quantities of ROS such as superoxide anion, hydrogen peroxide, nitric oxide, and their derivatives, which target the surrounding tissue and cause oxidative stress intra- or extracelluarly (Bautista et al. 1990; Sakaguchi 2004; Sakaguchi and Furusawa 2006). The intracellular components result in dysfunction of mitochondria or mitochondrial enzyme systems (Jaeschke et al. 2012).

On the other hand, in ischemia/reperfusion (I/R) model, it is well-known that ROS production and the subsequent secretion of TNF-α by inflammatory cells significantly contributes to the injury, and preconditioning by short-term ischemia increases resistance to subsequent lethal ischemia/reperfusion with the suppression of massive inflammatory activation. The protective mechanism of preconditioning involves the enhanced ROS-scavenging activity caused by the production of small amount of ROS by short-term ischemia (Morihira et al. 2006; Teoh et al. 2003).

In the previous paper, we reported that protective mechanisms mediated by proline supplementation in GalN-induced liver injury is attributable to the early regenerative response thorough the activation of NF-κB/IL-6/STAT3 pathway and early reduction of inflammation (Obayashi et al. 2012). We hypothesized that production of small amount of ROS in the mitochondria through the proline metabolism (Wu et al. 2011) may activate NF-κB and be attributable to these protective mechanisms, mimicking the protective mechanism of preconditioning model by short-term ischemia.

**Figure 1:**
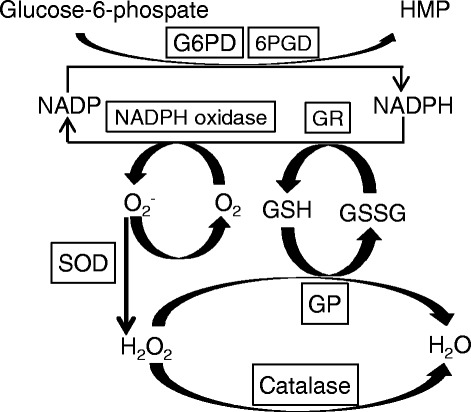
Primary ROS-eliminating system in the liver. NADPH-dependent production and elimination of ROS is shown. The abbreviation stands for as follows: GP, glutathione peroxidase; GR, glutathione reductase; GSH, reduced glutathione; GSSG, oxidized glutathione; SOD, superoxide dismutase; G6PD, glucose-6-phosphate dehydrogenase; 6PGD, 6-phosphogluconate dehyderogenase; HMP, hexose monophosphate pathway.

To further investigate the mechanism of the protective effect of proline on GalN-induced hepatitis, we investigated the effect of proline on the anti-oxidative system in the liver (Figure 1).

## Results

### The effect of proline on the activation of glutathione redox cycle

Glutathione redox cycle is one of the major pathways of antioxidant defense. In order to investigate the effect of proline on the activation of glutathione redox cycle in the liver, we examined the activity of GP, GR, the amount of glutathione, and the mRNA expression involved in glutathione synthesis in the liver.

**Figure 2:**
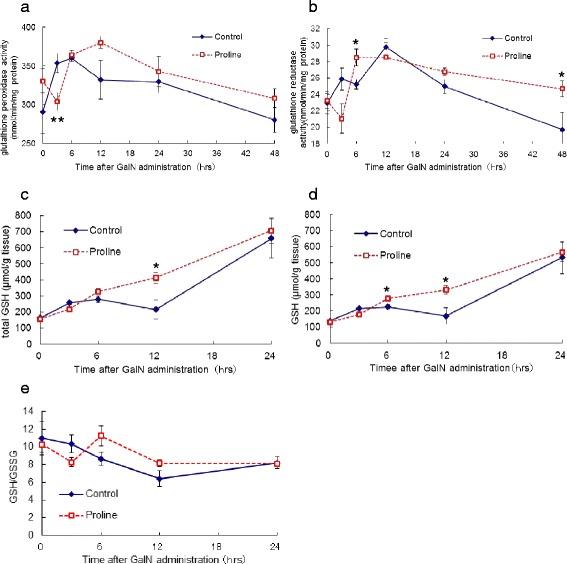
The activity of enzymes involved in glutathione redox cycle and the level of glutathione in the liver. The activity of GP **(a)**, GR **(b)** and the level of total glutathione **(c)**, GSH **(d)**, GSH/GSSG **(e)**. Enzyme activities and the level of glutathione were determined as described in Materials and methods. Results are mean ± SEM. Time course of control and proline groups are denoted by solid line and dotted line, respectively. A significant difference between the two groups is denoted by “asterisk” (*P < 0.05, **P < 0.01).

The activity of GP and GR dropped at 3 h after GalN administration in the proline group and the level was significantly lower compared with control group (GP activity: 353.4 ± 12.2 vs 303.8 ± 11.7 nmol/min/mg protein (P < 0.01), GR activity: 25.9 ± 1.3 vs 21.1 ± 1.8 nmol/min/mg protein (P = 0.06), control vs proline group) (Figure 2a,b). The activity of both GP and GR elevated afterwards with the significantly higher activity of GR at 6 h in the proline group (25.2 ± 0.6 vs 28.5 ± 0.1 nmol/min/mg protein (P < 0.05), control vs proline group) and with the higher activity of GP at 12 h (332.0 ± 25.0 vs 380.0 ± 7.6 nmol/min/mg protein (P = 0.10), control vs proline group).

The amount of total glutathione in the liver gradually increased in the proline group after GalN administration with levels two-times higher at 12 h relative to the control group (215.2 ± 60.7 vs 412.4 ± 32.9 μmol/g tissue (P < 0.05), control vs proline group) (Figure 2c). The amount of reduced glutathione (GSH) also gradually increased in the proline group with the significantly higher amount at 6 h (224.7 ± 14.7 vs 277.0 ± 16.8 μmol/g tissue (P < 0.05), control vs proline group) and 12 h (167.0 ± 52.8 vs 331.0 ± 27.4 μmol/g tissue (P < 0.05), control vs proline group) (Figure 2d).

**Figure 3:**
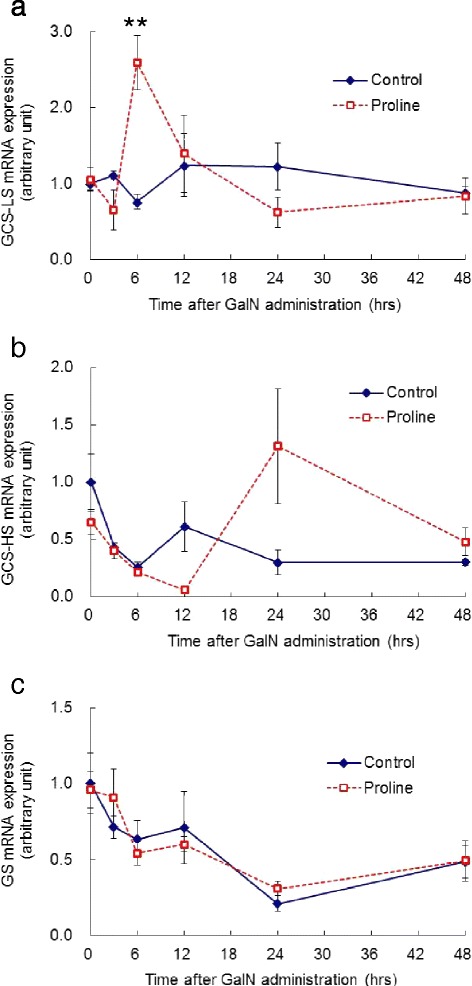
Hepatic expression of genes involved in glutathione synthesis. Hepatic expression of GCS-LS **(a)**, GCS-HS **(b)**, GS **(c)** mRNA after GalN administration. The level of mRNA is expressed as the expression level relative to the average for the control group at 0 h. Results are mean ± SEM. Time course of control and proline groups are denoted by solid line and dotted line, respectively. A significant difference between the two groups is denoted by “asterisk” (**P < 0.01).

The rate-limiting enzyme of glutathione synthesis, γ-glutamylcysteine synthetase (GCS), is composed of a catalytic (GCS-HS) and regulatory subunit (GCS-LS). Glutathione synthesis is also catalyzed by the second enzyme, glutathione synthetase (GS). Since glutathione synthesis is regulated by these enzymes, we examined the mRNA expression of these enzymes in the liver. Although the mRNA expression of GCS-HS and GS didn’t show significant difference between control and proline group at any time points, that of GCS-LS dramatically increased in the proline group at 6 h with the level three times higher (0.76 ± 0.10 vs 2.59 ± 0.36 (P < 0.01), control vs proline group) (Figure 3).

### The effect of proline on catalase activity

In addition to GP, catalase is the other major hydrogen-peroxide-detoxyfying enzyme, which converts hydrogen peroxide to water utilizing NADPH. In order to investigate the effect of proline on the antioxidant defense system in the liver, we examined the catalase activity at each time point before and after GalN administration.

**Figure 4:**
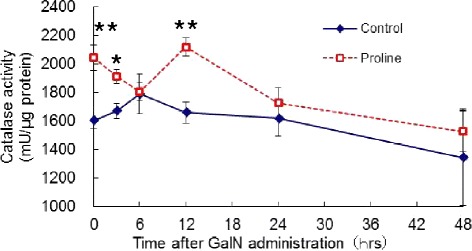
The activity of catalase in the liver after GalN administration. The activity of catalase was determined as described in Materials and methods. Results are mean ± SEM. Time course of control and proline groups are denoted by solid line and dotted line, respectively. A significant difference between the two groups is denoted by “asterisk” (*P < 0.05, **P < 0.01).

At 0 h, one hour after proline treatment and immediately before GalN injection, the levels of catalase activity was significantly higher in the proline group (1603 ± 63 vs 2042 ± 87 mU/μg protein (P < 0.01), control vs proline group) (Figure 4). Until 6 h, the catalase activity in the proline group gradually declined, although it was still significantly higher at 3 h than control group (1670 ± 53 vs 1910 ± 49 mU/μg protein (P < 0.05), control vs proline group). At 12 h, the catalase activity in the proline group elevated again with the significantly higher activity in proline group, despite of almost steady level in control group (1657 ± 75 vs 2116 ± 64 mU/μg protein (P < 0.01), control vs proline group).

## Discussion

To investigate the effect of proline on the ROS-eliminating system in the liver, we examined the activity of major anti-oxidative enzymes, i.e. catalase, GP, GR, and anti-oxidative substances which relate to the functions of those enzymes, i.e. glutathione. We found that catalase was already activated in proline group before GalN administration, and the activity was sustained at significantly higher level than control group up to 3 h after GalN injection. From this result, it can be assumed that proline administration itself causes oxidative stress in the liver, and it may be probably attributable to the metabolism of proline. There are a couple of possible metabolic reactions of proline which may produce ROS. One of the possibilities is the first enzyme involved in proline metabolism, proline oxidase, as it was already speculated in the previous report (Obayashi et al. 2012). The other possible mechanism is increased reactive oxygen production by the respiratory chain in mitochondria through the enhanced electron flow caused by pyruvate derived from proline (Marí et al. 2002).

At early phase of GalN hepatitis, i.e. at 3 h after GalN injection, although the total glutathione level began to increase in both groups, the activities of GR and GP in proline group were significantly lower in proline group than those in control group. From these results, it can be assumed that the glutathione redox cycle has not yet been enhanced in proline group relative to control group at 3 h. Taken together, at early phase of GalN hepatitis, enhanced catalase is the major hydrogen peroxide-detoxifying pathway in proline group, and its detoxifying capacity is higher compared with control group.

On the next phase, from 6 to 12 h after GalN ingestion, the GSH level in the liver of the proline group was significantly higher and the GSH/GSSG ratio in the proline group tended to be higher. In addition, GR activity was significantly higher at 6 h, and GP activity tended to be higher at 12 h. These results suggest that the glutathione redox cycle in proline group was significantly activated relative to control group from 6 to 12 h.

At 12 h, catalase activity was also upregulated and significantly higher in proline group and differences of total glutathione and reduced glutathione level between both groups was the most significant with levels two times higher in proline group. These results suggest that from 6 to 12 h after GalN injection, the ROS-eliminating system in the liver of proline group was significantly activated than that of control group, probably with the most significant difference at 12 h. Later than 24 h, catalase, GR and GP activity were still prone to be higher in proline group with the significantly higher activity of GR at 48 h.

Activation of glutathione redox system is very important because GP and GSH S-transferase reduce not only hydrogen peroxide but also organic peroxide. Furthermore, although hydrogen peroxide can also be reduced by catalase which is present only in the peroxisome, glutathione redox cycle is particularly important in the mitochondria, because there is no catalase (Fernández-Checa et al. 1997; Garcia-Ruiz and Fernandez-Checa 2006). In the present study, we found that the activity of Mn-SOD, which is known to be expressed only in the mitochondria, tended to be higher in proline group from 6 to 12 h (P = 0.13 at 6 h, P = 0.19 at 12 h, data not shown). The result strongly suggests the possibility that the ROS production in mitochondria increased at the time. The cooperative upregulation of glutathione redox system of the proline group should help to more effectively eliminate ROS in the mitochondria, relative to control group.

Glutathione is synthesized in all mammalian cells. Reduced glutathione (GSH) is the predominant form, whereas the GSSG content is less than 1% of GSH (Akerboom et al. 1982). Almost 90% of cellular GSH are in the cytosol, 10% is in the mitochondria and a small percentage is in the endoplasmic reticulum (Meredith and Reed 1982).

Glutathione is synthesized in cytosol and the synthetic process involves two ATP-requiring steps. The first step is catalyzed byγ-glutamylcysteine synthetase (GCL) and is considered to be rate limiting step. The second step is catalyzed by glutathione synthetase (GS). γ-Glutamylcysteine synthetase is composed of a heavy or catalytic (GCS-HS) and a light or modifier (GCS-LS) subunit. Modifier subunit is enzymatically inactive but plays an important regulatory function by lowering the Km of GCL for glutamate and raising the Ki for GSH (Huang et al. 1993a, b). In the present study, we found that regulatory subunit, GCL-LS mRNA was significantly upregulated in proline group at 6 h with the levels three times higher. Since there was no significant difference of the mRNA level of GCS-HS and GS between control and proline group at any time points, it can be assumed that the activity of GCS in proline group might be upregulated by the upregulation of regulatory subunit of GCS, leading to the significant upregluation of total GSH from 6 h to 12 h.

In addition to anti-oxidant function, it is reported that GSH plays an important role in cell proliferation. Lu et al. reported that when rat hepatocytes were plating under low density, which stimulates hepatocyte to shift from Go to G1 phase, increased GSH level with the activation of GCS (Lu and Ge 1992). It is also reported that after 2/3 partial hepatectomy, hepatic GSH increased due to increased biosynthesis prior to the onset of DNA synthesis (Huang et al. 1998). When this increase in GSH was blocked, liver regeneration was impaired (Huang et al. 2001). Furthermore, the increase of GSH directly correlates with the growth of liver cancer cells (Huang et al. 2001). In our previous study, we reported that the regenerative signaling, IL-6/STAT3 pathway, was strongly activated from 12 to 24 h after GalN injection followed with the significant upregulation of the marker of proliferation, histone H3 mRNA at 24 h in proline group. The significant activation of proliferative signaling in the liver of the proline group might be partially attributable to the early increase of GSH content.

**Figure 5:**
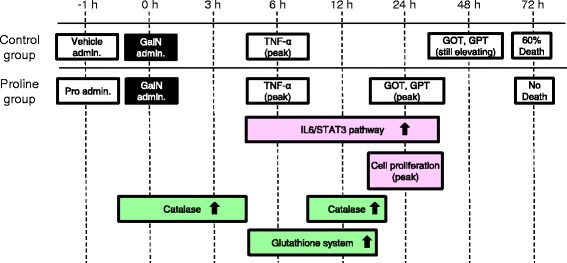
The elevation of the inflammatory indices, the proliferative signaling and the activation of the ROS-eliminating system. Outline of the results obtained so far. The level of inflammatory indices (TNF-α, GOT, GPT), the proliferative signaling (IL-6/STAT3 pathway), and the proliferative marker (histone H3) are investigated in the previous paper (Obayashi et al. 2012).

In the previous study, we also reported that plasma level of TNF-αpeaked at 6 h in both group and gradually decreased later than 12 h (Obayashi et al. 2012). On the other hand, the levels of GOT and GPT began to increase from 3 h in both groups, peaked at 24 h and decreased at 48 h in proline group, although those levels in control group continued to increase up to 48 h (Figure 5). These results suggest that the oxidative stress should be introduced at very early phase in the liver of both groups.

This study has demonstrated that the early introduction of anti-oxidative property in the liver of proline group must significantly contribute to protecting parenchymal and non-parenchymal cells from oxidative stress caused by GalN injection. It should be also noted that early upregulation of GSH in the liver of the proline group should plays an important role in the activation of proliferative signaling within 24 h. The low level production of ROS by metabolic process of proline may work as a preconditioning to effectively induce anti-oxidative system against the significant oxidative stress induced by GalN injection. As is recently suggested, proline can be considered as a “functional amino acids” (Wu 2010, 2014; Wu et al. 2013).

## Conclusions

This study demonstrated that proline supplementation in GalN-induced liver failure model significantly activated ROS-eliminating system such as catalase and glutathione system in the liver within 24 hours after GalN administration. It is thought that early induction of these antioxidative systems should significantly contribute to the protective mechanism of proline supplementation in this model.

## Materials and methods

### Animals and experimental design

Nine-week-old male Fischer 344 rats (Charles River Laboratory, Japan), weighing from 180-200 g, were maintained at 23°C, given standard laboratory chow and water ad libitum, and kept under a 12 h light (7:00-19:00)/12 h dark schedule. Inbred rat, Fischer 344, was chosen because of the large individual difference of liver damage induced by GalN in case of outbred rats. All animals received humane care in accordance with the Japanese guidelines for animal experimentation (Japanese Association for Laboratory Animal Science). All procedures used in animal experiments were approved by the Animal Ethics Committee of the institution. Before the start of the experiment, food was withdrawn for 15 h, but the rats had free access to a 10% glucose solution. The animals were divided to 2 groups, each of which consisted of 5 animals. The control group received a single intraperitoneal injection of 30% GalN saline solution between 9:00 and 10:00 am at a dose of 1.4 g/kg body weight, and the proline group received a 10% proline solution orally at a dose of 2 g/kg body weight 1 h before GalN administration. Pre-administration of proline was determined based on the report that it’s more effective than administration after GalN treatment (Ajinomoto Co., Inc. 1996).

After animals were killed by exsanguination at each time point, the livers were harvested and rinsed in ice-cold saline. A part of the liver was immediately frozen in liquid nitrogen for mRNA quantification.

### Preparation of the liver sample

For the measurement of glutathione, about 0.1 g of the rat liver was precisely weighed and homogenized with Polytron homogenizer in 1 ml of extraction buffer (5% sulfosalicylic acid, 5 mM EDTA) on ice. The homogenate was centrifuged at 15000 rpm at 0°C for 10 min, and 100 μl of the supernatant was diluted by 700 μl of MES buffer for sample solution.

For the measurement of catalase, about 0.1 g of the rat liver was homogenized with Polytron homogenizer in 0.8 ml phosphate buffer (50 mM potassium phosphate buffer, pH 7.5, 1 mM EDTA). The homogenate was centrifuged at 10000 rpm at 4°C for 15 min, and the protein concentration of the supernatant was quantified using BCA protein assay (Pierce, IL, USA).

For the measurement of glutathione reductase and peroxidase, glutathione was removed from the supernatant. Briefly describing, 450 μl phosphate buffer was added to 50 μl supernatant and was subject to ultrafiltration (ULTRAFREE-0.5 Centrifugal Filter Device (UFV5BGC100), Millipore, MA, USA) at 12000 × g at 4°C for 60 min. The retained fluid in the inner tube was filled up to about 100 μl by the phosphate buffer. The inner tube was rinsed with 100 μl phosphate buffer to combine with the retained fluid. The protein concentration of the sample was quantified using BCA protein assay.

### Enzyme assays

The activity of GP was assayed by Glutahione Peroxidase Assay Kit (Cayman Chemical, MI, USA) as described in manufacturer’s instructions. The supernatant of the liver homogenate was diluted to 0.3 mg/ml of protein concentration for the assay.

The activity of GR was assayed by Glutahione Reductase Assay Kit (Cayman Chemical, MI, USA) as described in manufacturer’s instructions. The supernatant of the liver homogenate was diluted to 2.0 mg/ml of protein concentration for the assay.

Catalase activity was assayed by Amplex® Red Catalase Assay Kit (Life Technologies, USA) as described in manufacturer’s instructions. The supernatant of the liver homogenate was diluted to 0.375 μg/ml of protein concentration for the assay.

### Standard curve of GSH and GSSG

The standards of GSH and GSSG were prepared and diluted in extraction buffer (5% sulfosalicylic acid, 5 mM EDTA): MES buffer (0.4 M 2-(N-morpholino) ethanesulphoric acid, 0.1 M sodium phosphate, 2 mM EDTA, pH 6.5) = 1:15 or 1:7 respectively to provide 10 concentrations in the rage of 0.5 – 32 μM. Fifty microliters of standard solutions were mixed with assay cocktail (12.5 μl of 4 mM NADPH in 5% NaHCO_3,_ 25 μl of GSH reductase solution (Roche, No.105678, 5 mg/ml), 12.5 μl of 10 mM 5,5′-dithiobis-2-nitrobenzoic acid in 0.1 M phosphate buffer, 75 μl MES buffer, 25 μl distilled water) on 96-well plate. The absorbance at 405 nm was monitored at every 30 sec for 10 min at 37°C to provide calibration curve. From the changes in absorbance over time for standards, the concentration of unknown samples were calculated.

### Analysis of total glutathione and GSSG

For total glutathione determination, 100 μl of sample solution was diluted by 150 μl of MES buffer for further analysis. For GSSG determination, 500 μl of sample solution was added 5 μl of 1 M 2-vinylpyridine in ethanol, mixed and incubated at room temperature for 60 min to mask the GSH.

Fifty microliters of the above diluted or 2- vinylpyridine -treated sample solution was mixed with assay cocktail on 96-well plate. The absorbance at 405 nm was monitored at every 30 sec for 10 min at 37°C. The amount of GSH was calculated as the difference between total glutathione and GSSG.

### Isolation of total RNA and cDNA synthesis

Total RNA was isolated from liver tissue with ISOGEN (Nippon Gene Co., Ltd., Tokyo, Japan) according to the manufacturer’s instructions. Total RNA was used as the template for cDNA synthesis with Superscript II reverse transcriptase (Invitrogen, CA, USA).

### Real-Time PCR

The specificity of the PCR amplification with each primer pair was electrophoretically confirmed with a 4% NuSieve 3:1 agarose gel (Cambrex Corporation, USA). The PCR reactions were carried out in 20-μl reaction volumes using the SYBR Green PCR Master Mix (Applied Biosystems, USA) with 600 nM oligonucleotide primers and cDNA reverse-transcribed from 10 ng or 40 ng total RNA. For signal detection, the ABI Prism 7700 sequence detector (Life Technologies, CA, USA) was programmed to execute an initial step of 2 min at 50°C and 10 min at 95°C, followed by 40 thermal cycles of 15 sec at 95°C and 1 min at 60°C. The amount of the target gene was determined using a calibration curve that was constructed using serial dilutions of the target gene. The level of mRNA was expressed as the expression level relative to the average for the control group at 0 h, which was set to 1.0.

### Expression of data and statistical analysis

The results are expressed as the mean ± SEM. The Student’s *t* test was used for the comparison of data from two groups. The difference between groups was considered significant when P was less than 0.05.

## Abbreviations

GalN: D-galactosamine
GP: Glutathione peroxidase
GR: Glutathione reductase
GSH: Reduced glutathione
GSSG: Oxidized glutathione
GCS: γ-glutamylcysteine synthetase
GCS-HS: Catalytic (heavy) subunit ofγ-glutamylcysteine synthetase
GCS-LS: Regulatory (light) subunit ofγ-glutamylcysteine synthetase
SOD: Superoxide dismutase
IL-6: Interleukin-6
STAT3: Signal transducer and activator of transcription 3
TNF-α: Tumor necrosis factor-α
NF-κB: Nuclear factor kappa B
GOT: Glutamic oxaloacetic transaminase
GPT: Glutamic pyruvic transaminase
ROS: Reactive oxygen species
NOS: Nitric oxide synthase
G6PD: Glucose-6-phosphate dehydrogenase
6PGD: 6-phosphogluconate dehyderogenase
HMP: Hexose monophosphate pathway

## References

[CR1] Akerboom TP, Bilzer M, Sies H (1982). The relationship of biliary glutathione disulfide efflux and intracellular glutathione disulfide content in perfused rat liver. J Biol Chem.

[CR2] Bautista AP, Mészáros K, Bojta J, Spitzer JJ (1990). Superoxide anion generation in the liver during the early stage of endotoxemia in rats. J Leukoc Biol.

[CR3] Fernández-Checa JC, Kaplowitz N, García-Ruiz C, Colell A, Miranda M, Marí M, Ardite E, Morales A (1997). GSH transport in mitochondria: defense against TNF-induced oxidative stress and alcohol-induced defect. Am J Physiol.

[CR4] Garcia-Ruiz C, Fernandez-Checa JC (2006). Mitochondrial glutathione: hepatocellular survival-death switch. J Gastroenterol Hepatol.

[CR5] Grün M, Liehr H, Rasenack U (1977). Significance of endotoxaemia in experimental “galactosamine-hepatitis” in the rat. Acta Hepatogastroenterol (Stuttg).

[CR6] Huang CS, Anderson ME, Meister A (1993). Amino acid sequence and function of the light subunit of rat kidney gamma-glutamylcysteine synthetase. J Biol Chem.

[CR7] Huang CS, Chang LS, Anderson ME, Meister A (1993). Catalytic and regulatory properties of the heavy subunit of rat kidney gamma-glutamylcysteine synthetase. J Biol Chem.

[CR8] Huang ZZ, Li H, Cai J, Kuhlenkamp J, Kaplowitz N, Lu SC (1998). Changes in glutathione homeostasis during liver regeneration in the rat. Hepatology.

[CR9] Huang ZZ, Chen C, Zeng Z, Yang H, Oh J, Chen L, Lu SC (2001). Mechanism and significance of increased glutathione level in human hepatocellular carcinoma and liver regeneration. FASEB J.

[CR10] Jaeschke H, McGill MR, Ramachandran A (2012). Oxidant stress, mitochondria, and cell death mechanisms in drug-induced liver injury: lessons learned from acetaminophen hepatotoxicity. Drug Metab Rev.

[CR11] Jirillo E, Caccavo D, Magrone T, Piccigallo E, Amati L, Lembo A, Kalis C, Gumenscheimer M (2002). The role of the liver in the response to LPS: experimental and clinical findings. J Endotoxin Res.

[CR12] Lu SC, Ge JL (1992). Loss of suppression of GSH synthesis at low cell density in primary cultures of rat hepatocytes. Am J Physiol.

[CR13] MacDonald JR, Beckstead JH, Smuckler EA (1987). An ultrastructural and histochemical study of the prominent inflammatory response in D(+)-galactosamine hepatotoxicity. Br J Exp Pathol.

[CR14] Marí M, Bai J, Cederbaum AI (2002). Toxicity by pyruvate in HepG2 cells depleted of glutathione: role of mitochondria. Free Radic Biol Med.

[CR15] Meredith MJ, Reed DJ (1982). Status of the mitochondrial pool of glutathione in the isolated hepatocyte. J Biol Chem.

[CR16] Morihira M, Hasebe N, Baljinnyam E, Sumitomo K, Matsusaka T, Izawa K, Fujino T, Fukuzawa J, Kikuchi K (2006). Ischemic preconditioning enhances scavenging activity of reactive oxygen species and diminishes transmural difference of infarct size. Am J Physiol Heart Circ Physiol.

[CR17] Obayashi Y, Arisaka H, Yoshida S, Mori M, Takahashi M (2012). Proline protects liver from D-galactosamine hepatitis by activating the IL-6/STAT3 survival signaling pathway. Amino Acids.

[CR18] Sakaguchi S (2004). Metabolic aspects of endotoxin as a model of septic shock–approached from oxidative stress. Yakugaku Zasshi.

[CR19] Sakaguchi S, Furusawa S (2006). Oxidative stress and septic shock: metabolic aspects of oxygen-derived free radicals generated in the liver during endotoxemia. FEMS Immunol Med Microbiol.

[CR20] Teoh N, Leclercq I, Pena AD, Farrell G (2003). Low-dose TNF-alpha protects against hepatic ischemia-reperfusion injury in mice: implications for preconditioning. Hepatology.

[CR21] Wu G (2010). Functional amino acids in growth, reproduction, and health. Adv Nutr.

[CR22] Wu G (2014). Dietary requirements of synthesizable amino acids by animals: a paradigm shift in protein nutrition. J Anim Sci Biotechnol.

[CR23] Wu G, Bazer FW, Burghardt RC, Johnson GA, Kim SW, Knabe DA, Li P, Li X, McKnight JR, Satterfield MC, Spencer TE (2011). Proline and hydroxyproline metabolism: implications for animal and human nutrition. Amino Acids.

[CR24] Wu G, Wu Z, Dai Z, Yang Y, Wang W, Liu C, Wang B, Wang J, Yin Y (2013). Dietary requirements of “nutritionally non-essential amino acids” by animals and humans. Amino Acids.

